# 

**Published:** 1897-04

**Authors:** 


### CONTENTS.

ORIGINAL COMMUNICATIONS—

EARLY DIAGNOSIS AND VAGINAL HYSTERECTOMY IN CANCER OF THE UTERUS. By J. A. Goggans, M.D., Alexander City, Ala. .... 73
ON THE TREATMENT OF ACNE. By William S. Gottheil, M.D., New York. .... 77
SOME PRACTICAL THOUGHTS ON THE DEVELOPMENT OF THE HUMAN RACE AND OBSTETRIC NURSING. By R. R. Kime, M.D., Atlanta, Ga. .... 83
THE THERAPEUTIC APPLICATION OF CHLOROFORM IN LABOR. By John N. Upshur, M.D., Richmond, Va. .... 89
NEPHRECTOMY FOR LARGE MULTIPLE ABSCESS OF THE KIDNEY. By William C. Duggan, M.D., Louisville, Ky. .... 93
SEBACEOUS TUMORS AND THEIR CURE. By A. Bethune Patterson, M.D., Atlanta, Ga. 94
ANEMIA AND ITS TREATMENT. By L. A. Felder, M.D., Atlanta, Ga. .... 95

SOCIETY REPORTS—

ATLANTA SOCIETY OF MEDICINE .... 98

CORRESPONDENCE—

NEW YORK LETTER .... 106

EDITORIAL—

WILL IT COME TO THIS? .... 109
MEDICAL ASSOCIATION OF GEORGIA .... 112
COLLEGE COMMENCEMENTS .... 113

SELECTIONS AND ABSTRACTS—

OPHTHALMOLOGY AND OTOLOGY .... 116
MISCELLANEOUS .... 121

MEDICAL ITEMS .... 139
BOOK REVIEWS .... 143
MEDICAL ITEMS—CONTINUED .... x, xii, xiv, xxvi
ADVERTISERS' INDEX TO ADVERTISEMENTS .... xviii

